# Post-Modified Polypeptides with UCST-Type Behavior for Control of Cell Attachment in Physiological Conditions

**DOI:** 10.3390/ma11010095

**Published:** 2018-01-09

**Authors:** Xuan Xue, Lalitha Thiagarajan, James E. Dixon, Brian R. Saunders, Kevin M. Shakesheff, Cameron Alexander

**Affiliations:** 1 School of Pharmacy, the University of Nottingham, University Park, Nottingham NG7 2RD, UK; scarlett.xue@nottingham.ac.uk (X.X.); Lalitha.Thiagarajan@nottingham.ac.uk (L.T.); James.Dixon@nottingham.ac.uk (J.E.D.); 2 School of Materials, the University of Manchester, Manchester M13 9PL, UK

**Keywords:** UCST polymer, thermo-responsive polypeptides, controlled cell attachment

## Abstract

Upper Critical Solution Temperature (UCST)-type thermally responsive polypeptides (TRPs) with phase transition temperatures around 37 °C in phosphate-buffered saline (PBS) buffer (pH 7.4, 100 mM) were prepared from poly(l-ornithine) hydrobromide and coated on non-tissue culture-treated plastic plates (nTCP). Cell adhesion was observed at temperatures above the phase transition temperature of the coating polymer (39 °C), while cell release was triggered when the culture temperature was switched to 37 °C. Approximately 65% of the attached cells were released from the surface within 6 h after changing the temperature, and more than 96% of the released cells were viable. Water contact angle measurements performed at 39 and 37 °C demonstrated that the surface hydrophobicity of the new TRP coatings changed in response to applied temperature. The cell attachment varied with the presence of serum in the media, suggesting that the TRP coatings mediated cell attachment and release as the underlying polymer surface changed conformation and consequently the display of adsorbed protein. These new TRP coatings provide an additional means to mediate cell attachment for application in cell-based tissue regeneration and therapies.

## 1. Introduction

Controlled delivery of specific cells to correct defective or damaged tissue has become a significant research goal in tissue regenerative therapies. However, design of supports to promote cell attachment is highly complex and the determinants of cell adhesion range from surface charge [1,2], hydrophilic/hydrophobic balance [1,3,4,5,6,7], functional group type and content [1,7,8,9], to surface roughness and topology [10,11]. For cell culture and delivery applications, it is usually important to generate materials which exhibit high cellular affinity during the cell growth and transport stages, but low affinity when the cells are in their desired site of action or expansion. It is also desirable to be able to switch on or off these cell surface interactions via a simple stimulus, as traditional cell harvesting methods (e.g., high shear or proteases) are not applicable in vivo.

Synthetic polymers have many properties which can be systematically altered, and thus have advantages over natural polymers when tuning of function is required. Certain polymers exhibit changes of solubility in aqueous solutions in response to changes in temperature, and those materials with Lower Critical Solution Temperature (LCST)-type phase transitions, have been widely studied for tissue engineering [12,13], drug delivery [14,15], and cellular binding [8,9,16]. However, there has been relatively little work focused on polymers with Upper Critical Solution Temperature (UCST)-type phase transition behavior for these applications. The limited evaluation of UCST transitions in practical applications to date is possibly because only a few polymers exhibit UCST behavior in aqueous media [17,18,19,20,21]. PNAGAm (poly(*N*-acryloyl glycinamide)) and related materials (e.g., poly(*N*-acryloylasparaginamide), PNAAAm) have been the most studied reversible hydrogen bonding based UCST polymers, as a result of their phase transitions being adjustable to body temperature in ionic solutions [19,20,22,23]. For biomedical applications, examples have been demonstrated for PNAGAm based hydrogels as thermo-responsive drug delivery systems [24], but not for controllable cell culture and delivery. However, we have recently shown that variants of PNAGAm based polymer brushes may be suitable for applications in tissue engineering and regenerative medicine through temperature-controlled cell attachment [25]. On the other hand, of particular note is the controlled UCST-type behavior for ureido-modified polypeptide poly(l-ornithine)-*co*-poly(l-citrulline) (POC) under physiological conditions, the transition temperature of which can be adjusted up to 31 °C by ureido content [26]. Importantly, modified polypeptides of this type potentially offer the advantages of biocompatibility, biodegradability and cellular affinity, allowing them to be good candidate materials for cell delivery.

Here, we report the synthesis and characterization of polypeptides with UCST-type behavior, utilizing ureido and methyl isocyanate modification of poly(l-ornithine) hydrobromide. The resultant materials showed reversible phase separation from phosphate-buffered saline (PBS) buffer (pH 7.4, 100 mM) at physiological temperatures. The polymer was adsorbed from solution to coat non tissue culture treated plastic plate (nTCP) at its transition temperature (39 °C). The resultant coating gave similar 3T3 cell attachment at 39 °C to uncoated cell culture treated plastic, while cell detachment was triggered by a temperature change to 37 °C. Approximately 65% of attached cells were released within 6 h in response to the temperature change and more than 96% of the released cells were viable at all tested time points.

## 2. Results

### 2.1. Synthesis and Characterization of Modified Polypeptides

**Figure 1:**
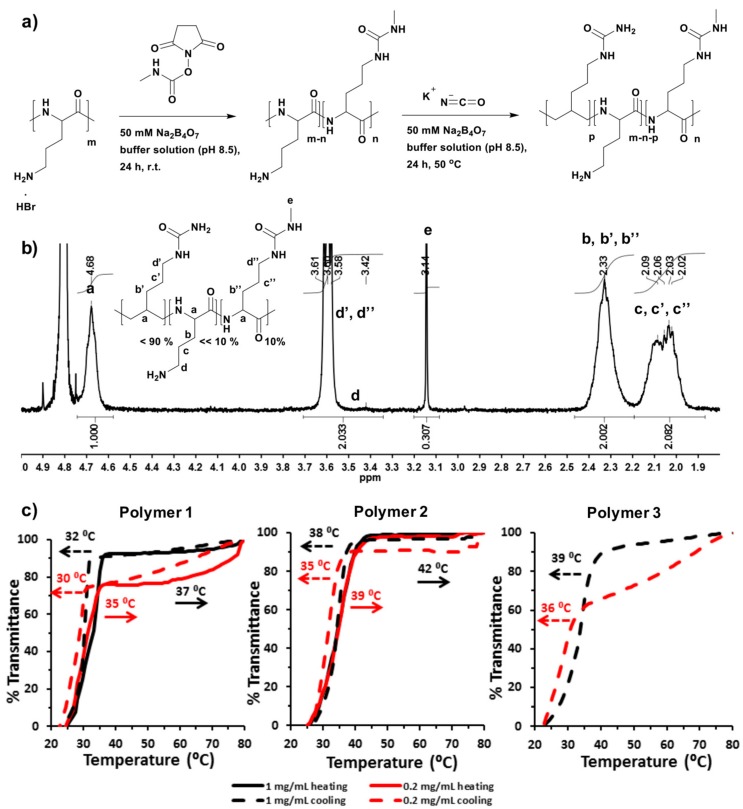
(**a**) Synthesis route of *N*-succinimidyl *N*-methylcarbamate modified poly(l-ornithine)-*co*-poly(l-citrulline) (POC) produced by a two-step reaction; (**b**) ^1^H-NMR (400 Hz, D_2_O) spectrum of Polymer **3** recorded at 70 °C; (**c**) Absorbance curves of Polymer **1**, Polymer **2** and Polymer **3** in 100 mM PBS buffer (pH 7.4). Transmittance at 500 nm of thermally responsive polypeptides (TRPs) solutions were measured at a scanning rate of 1 °C from 25 to 80 °C, and reverse experiments from 80 to 25 °C. Two polymer concentrations (1 mg/mL and 0.2 mg/mL) were measured.

Poly(l-ornithine)-*co*-poly(l-citrulline) (POC) was synthesized in a mixture of potassium cyanate (1.2 eq. molar ratio to ornithine) and poly(l-ornithine) hydrobromide in imidazole buffer solution (1 M, pH 7.0) according to a previous report [26]. The reaction contents were maintained with stirring at 50 °C, which is higher than the phase transition temperatures of all the target polymers in this study, to avoid polymer phase separation during the reaction time. The POC product was characterized by ^1^H-NMR and ^13^C-HSQC spectroscopy (400 Hz, D_2_O at 70 °C). The actual ureido modification ratio was determined from the NMR spectrum for Polymer **1**. (Figure S1, and Table 1) The modified POCs were prepared by reacting 10% of the amino groups of poly(l-ornithine) with *N*-succinimidyl *N*-methylcarbamate (as a safe alternative to methyl isocyanate) and then conversion of part of the rest amino groups to ureido groups was performed using potassium cyanate (Figure 1a). The *N*-succinimidyl *N*-methylcarbamate modification was completed by stirring at room temperature for 24 h to form a non-thermoresponsive water soluble intermediate product in buffer solution. The ureido modification was then started by direct addition of potassium cyanate to the same solution with an increase in the reaction temperature to 50 °C. Sodium tetraborate buffer solution (50 mM, pH 8.5) was employed in this one-pot two-step reaction to deprotonate amino groups. The modified POC product was characterized by ^1^H-NMR spectroscopy (400 Hz, D_2_O at 70 °C). (Figure S2 for Polymer **2** and Figure 1b for Polymer **3**, and Table 1) δ (ppm): 4.68 (polypeptide backbone: NHC*H*CO), 3.58–3.61 (methyl urea or ureido modification: CH_2_C*H*_2_NHCO), 3.42 (amino residue: CH_2_C*H*_2_NH_2_), 3.14 (methyl urea: NHC*H*_3_), 2.33 (NHCH*CH*_2_CH_2_CH_2_) and 2.02–2.09 (NHCHCH_2_C*H*_2_CH_2_). The actual grafting percentage of methyl urea was calculated by comparing the integral of the methyl signal (peak e to that of polypeptide backbone signal (peak a). The corresponding grafting percentage of ureido functionality was determined through first calculating the total actual ratio of methyl urea and ureido groups via comparing the integral of methylene signal (from both methyl urea and ureido modification, peak d’ and d’’) to that of the polypeptide backbone signal, and then subtracting the ratio contributed from the methyl urea modification. The actual percentage of amino residue was difficult to calculate directly from ^1^H-NMR spectra, and therefore was evaluated by subtracting the mol.% of methyl urea and ureido modification from 100% (Table 1). The number of amino residues was very small in Polymer **3**, so that peak (d) assigned to methylenes adjacent to amino groups was difficult to detect clearly in the NMR spectrum. Accordingly, the structure of Polymer **3** was further characterized by 2D ^13^C-HSQC spectrum (400 Hz, D_2_O at 70 °C). (Figure S3) δ (ppm): 57 (polypeptide backbone: NH*C*HCO), 40 (methyl isocyanate or ureido modification: CH_2_*C*H_2_NHCO), 28 (NHCH*C*H_2_CH_2_CH_2_) and 26 (NHCHCH_2_*C*H_2_CH_2_).

In addition, poly(ethylene glycol)-grafted-poly(l-lysine) (PEG-*g*-PLL) was synthesized by addition of 0.9 eq. (molar ratio to amino groups) of mPEG-*NHS* to PLL in buffer solution according to the literature [27]. 50 mM of sodium tetraborate buffer solution (pH 8.5) was used to deprotonate the amino groups of lysine. The reaction was completed within 6 h at room temperature. The polymers were purified by dialysis against water and then lyophilized. The PEG-*g*-PLL product was characterized by ^1^H-NMR spectroscopy (400 Hz, D_2_O). The actual grafting percentage of PEG was determined from the NMR spectrum (Figure S4 for Polymer **4** and Table 1).

The phase separation behaviors of the TRPs were analyzed in PBS buffer (100 mM, pH 7.4) under conditions similar to those in physiological media. In this work, the phase transition temperature (*T*_p_) was defined as an initial point temperature where transmittance first begins to drop. The *T*_p_ for Polymer **1** (at 1 mg/mL) was observed to be at 37 °C when heating and at 32 °C in cooling. Polymer **1** has 90% ureido groups and 10% amino residues. (Figure 1c, Polymer **1**, black line, and Table 1) and displayed the highest *T*_p_ as analogous with a previous report [26]. In more diluted solution (0.2 mg/mL), Polymer **1** *T*_p_ decreased by 2 °C compared to the value determined in higher concentration in both heating and cooling measurements (Figure 1c, Polymer **1**, red line, and Table 1). *N*-Succinimidyl *N*-methylcarbamate modification of PLO (Poly(l-ornithine)) alone did not generate a polymer with a UCST type phase transition. However, *N*-succinimidyl *N*-methylcarbamate combined with ureido modification resulted in an increase in *T*_p_ in both concentrations. The formation of methyl urea side-chains slightly increased the overall hydrophobicity of the polymer, and therefore increased the *T*_p_ [17]. Therefore, the phase separation for Polymer **2** was observed at 42 °C for higher concentration, and at 39 °C for a more diluted solution, with values 4–5 °C higher than those of Polymer **1** measured in the same conditions. Again, hysteresis were observed in the cooling measurements (Δ*T*_p_ = 4 °C for Polymer **2**, Figure 1c, and Table 1). In both Polymer **2** and **3**, the methyl urea ratio was kept to be 10%, and the ureido ratio was between 80% and 90%, leaving fewer than 10% amino groups left. However, according to NMR data (Figure 1b and Figure S2, and Table 1), the number of amino residues in Polymer **3** was less than Polymer **2**, and thus the number of ureido moieties in Polymer **3** was slightly more. This small difference in polymer structure is the reason why Polymer **3** was more hydrophobic, and its *T*_p_ could not be measured accurately during heating. Interestingly, the phase separation of Polymer **3** upon cooling was at 39 °C for 1 mg/mL and 36 °C for 0.2 mg/mL solution, i.e., temperatures that were at only 1 °C higher than those of Polymer **2** (Figure 1c, Polymer **3**, and Table 1). It was thus demonstrated that the *T*_p_ of all these polymers were concentration dependent, and that phase separations were evident at lower temperature in diluted solution.

### 2.2. Preparation and Characterization of Polymer Coated Substrates

The protocol used here to coat polymers onto the non-tissue culture treated plastic plate (nTCP) wells was modified from that used for coating PLO as recommended by the manufacturers. The concentration of all the polymer solution was 1 mg/mL in deionized H_2_O. Solutions of Polymer **1**, **2**, and **3** were heated (~50 °C) until solutions were completely clear, and then filtered through a 0.2 μm membrane to ensure sterility. This solution was added immediately to the wells. For 24-well plates, 300 μL of this solution was added to fully cover the well. The handling time was kept short, and the plate was incubated overnight at 39 °C (all the polymer solutions were transparent at this temperature), in order to avoid phase separation during the coating time. The supernatants were removed from the wells after incubation. The wells were rinsed with PBS to remove the incompletely adsorbed polymers from the surfaces. The coated plate was dried in air in the fume hood, and was stored at room temperature.

**Figure 2:**
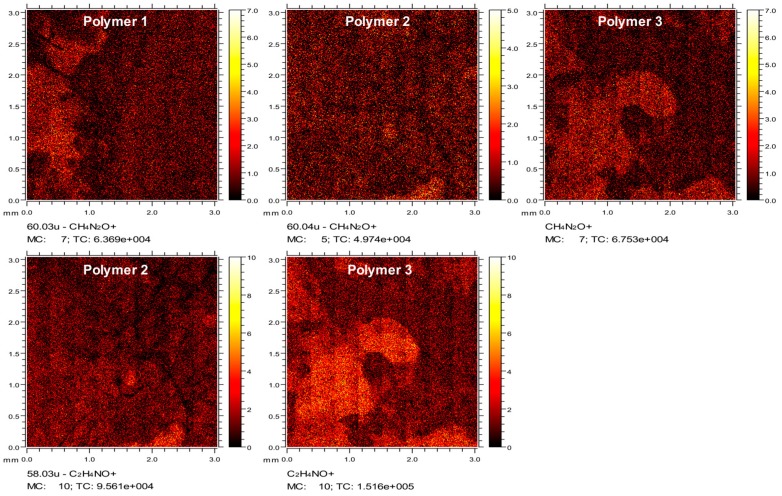
ToF-SIMS of polymer coated plastic coverslips. Images of secondary ion CH_4_N_2_O^+^ of ureido groups on Polymer **1**, **2**, and **3**-coated surfaces and the secondary ion C_2_H_4_NO^+^ from methyl urea substitution on Polymer **2** and **3**-coated surfaces. Please provide a high-resolution figure.

To confirm successful coating, polymers were attached onto plastic coverslips using the same protocol. Mass spectral analyses were used to characterize ions specific to coated surface chemistries. Time of Flight Secondary Ion Mass Spectrometry (ToF-SIMS) data demonstrated a clearly distinguishable CH_4_N_2_O^+^ peak due to ureido modification for Polymer **1**, **2** and **3**, which was negligible for uncoated and PLO, PLL or Polymer **4**-coated surfaces. The thin layer distribution of CH_4_N_2_O^+^ was seen in the ToF-SIMS images. (Figure 2a) Additional secondary ions were identified in the spectra that reinforced the identification of ureido modification, including CH_2_NO^+^ and CNO^−^ (Figure S5a). Polymer **2** and **3**-coated surfaces showed C_2_H_4_NO^+^ and CH_6_N^+^ peaks from the methyl urea formed by *N*-succinimidyl *N*-methylcarbamate modification, which was not observed in other surfaces. (Figure 2a and Figure S5b). Also, images showed the distribution of the thin layer for ion C_2_H_4_NO^+^ in Polymer **2** and **3** coated surfaces (Figure 2a). Again, the successful coating of Polymer **4**, with distinct peaks C_5_H_2_O_2_^−^ and C_5_H_3_O_3_^−^ assigned to the PEG modification, was also confirmed by ToF-SIMS (Figure S5c).

### 2.3. Screening Coating Polymers for Cell Attachment

**Figure 3:**
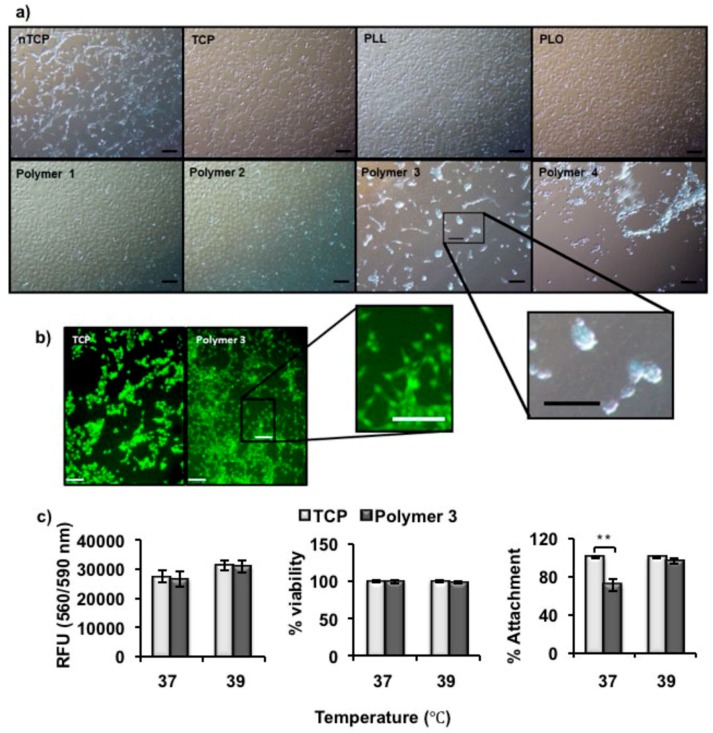
(**a**) Microscopy images of 3T3 cells taken after 24 h of culture on polymer-coated and -uncoated surfaces at 37 °C. Polymer **3** and Polymer **4**-coated nTCP were observed to be cell repellent at this screening temperature; (**b**) Microscope images of phalloidin-stained 3T3 cells taken after 24 h culture on uncoated TCP (Pos. Ctrl.) and Polymer **3** coated nTCP at 39 °C. Polymer **3**-coated surfaces showed a comparable number of cells attached at this temperature to that of Pos. Ctrl. (black scale bar = 100 µm, white scale bar = 150 µm); (**c**) The metabolic activity (left), percentage of viability (middle) and percentage of attachment of 3T3 cells measured after 24 h culture on either TCP or Polymer **3**-coated nTCP at 37 and 39 °C, respectively (*n* = 3). Statistical significance was determined using one-way analysis of variance (ANOVA), α = 0.05; * *p* ≤ 0.05; ** *p* ≤ 0.005. Error bars indicate standard deviation (SD).

The murine fibroblast cell line NIH-3T3 was used to assess the effect of polymer coated surfaces at various temperatures for cell attachment. As mentioned above, Polymer **1**, **2**, and **3** showed UCST-type behavior, with indicative *T*_p_ values of 32, 38 and 39 °C in PBS, respectively. Non-thermally responsive polymers such as PLL and PLO was used as controls for temperature dependent cell release. TCP and uncoated nTCP were used as the positive and negative controls for cell attachment. 3T3 cells (1 × 10^5^ cells per well) were seeded onto coated and uncoated wells and incubated at 37 °C overnight. Maximum cell attachment was observed in TCP followed by PLL, PLO, Polymer **1** and **2** coated nTCP, whereas fewer cells were attached in Polymer **3** coated in nTCP. It was also observed that cells preferred to aggregate before attaching to Polymer **3** coated surfaces at 37 °C. Poor cell attachment was observed in Polymer **4** coated nTCP. (Figure 3a) Floating cell aggregates could be observed in Polymer **4** coated wells, and very few cells were actually attached to the surface. We attribute this to the existence of hydrophilic PEG spacers on the surface, as expected. Therefore, Polymer **3** was the only thermally-responsive polymer that showed cell repulsive properties at body temperature (37 °C). Cell attachment was also observed in uncoated nTCP (negative control) surfaces upon longer incubation, due to the high inherent attachment ability of 3T3 cells.

**Figure 4:**
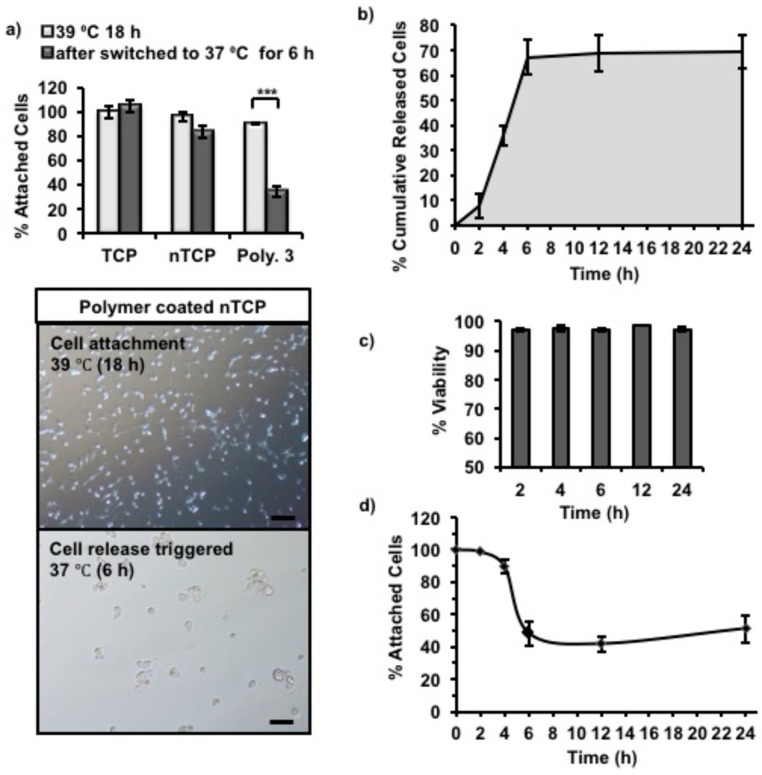
3T3 cell release from Polymer **3**-coated nTCP was triggered by switching incubation temperature from 39 °C to 37 °C. (**a**) The column graph showed the percentage of attached cells on Polymer **3**-coated nTCP, uncoated nTCP (Neg. Ctrl.) and uncoated TCP (Pos. Ctrl.) before and after cell release (6 h). Microscopy images of attached 3T3 cells on Polymer **3** coated nTCP were taken at the same time points (scale bar = 100 µm). Statistical significance was determined using one-way ANOVA, α = 0.05; * *p* ≤ 0.05; ** *p* ≤ 0.005; *** *p* ≤ 0.001. Error bars indicate standard deviation (SD). (**b**) The percentage of cumulative released cells with time was calculated from the results of CyQUANT assay; (**c**) The percentage of viable cells in the total number of released cells was calculated with the data obtained from Trypan blue assay; (**d**) The percentage of remaining attached cells on the surface at fixed intervals was calculated from Presto blue assay data (*n* = 3 independent experiments were performed in duplicate wells).

The initial data suggested that Polymer **3** is the most suitable candidate as a coating material that might repel cells from adhesion at body temperature and promote cell attachment at a different temperature. As the phase transition temperature of Polymer **3** (1 mg/mL) in PBS was 39 °C, we wanted to assess cell attachment at this temperature. It has been shown before that most cell types can survive and maintain a fairly constant growth rate and metabolic activity at 39 °C [28,29]. As shown in Figure 3b microscope images of Phalloidin stained cells, the number of cells adhering onto Polymer **3** coated surface was comparable to that imaged on TCP (positive control) after 24 h of culturing at 39 °C. The metabolic activity, percentage of metabolically active cells and attachment of the 3T3 cells were then further quantified by Presto Blue assays, Trypan Blue assays and manual counts from microscopy images. No significant change in metabolic activity was observed between cells cultured on TCP and on Polymer **3** coated plates. Metabolic activity was slightly higher at 39 °C. It has been shown that metabolic activity of cells increases with temperature until cell-damaging temperatures over 40 °C [30]. Furthermore, the attached cells were trypsinized and counted using Trypan Blue to assess viability. It was found that, for these three parameters, the behaviors of 3T3 cells on Polymer **3** were similar to those on TCP in most of the cases after 24 h incubation at 37 and 39 °C, respectively. However, the cell attachment on Polymer **3** (71 ± 6.2%) was much lower than that on TCP (96 ± 2.8%) after 24 h incubation at 37 °C. (Figure 4c) This was in accord with the observation from microscopy (Figure 3a, Polymer **3**) that cells did not properly adhere on Polymer **3** coated surfaces at 37 °C and preferred to aggregate before attaching.

### 2.4. Temperature Triggered Cell Release

Cells cultured on Polymer **1**, **2**, and **3**-coated nTCP at 39 °C were transferred to 37 °C to assess the temperature dependent cell releasing ability of the polymers. Among the three polymers, only Polymer **3** showed the temperature-dependent cell release, and hence it was investigated further. Initially, 3T3 cells were cultured on Polymer **3**-coated nTCP at 39 °C for 18 h, which gave sufficient time for cells to attach. The cell release was then triggered by switching the culture temperature from 39 to 37 °C. In Figure 4a, the percentage of attached cells on Polymer **3**-coated nTCP was compared to those on uncoated nTCP (Negative control) and TCP (Positive control). The cell attachment on Polymer **3** coated nTCP after incubation at 39 °C for 18 h was 90 ± 1% of the total number of cells seeded, which was slightly lower than the control surfaces (TCP: 100 ± 5%, nTCP: 96 ± 3.8%). After switching the incubation temperature to 37 °C for 6 h, only 34 ± 4% of the initially attached cells remained on the Polymer **3** coated surface. In Figure 4a, the microscope images showed the differences in cell attachment on Polymer **3** coated nTCP at these two time points. It can be seen from the images that right before cell release was triggered (cell attachment at 39 °C for 18 h) most of the cells were attached on the coated surfaces without showing significant cell aggregation. The same can be observed in the Live/Dead stained cells in Figure S6. After cell release was triggered for 6 h at 37 °C, fewer cells were observed to stay on the surface, but the remaining attached cells were spread out. Incorrect citation order. 

The cells released from the Polymer **3** surfaces after the temperature change were collected at specific times points (2, 4, 6, 12, and 24 h). As the cells aggregate after detaching from the polymer surface, the cells were individualized using trypsin and viability was determined using Trypan blue. To cross validate the exact cell numbers, the released cells were quantified in a plate reader using CyQUANT NF dye. The cumulative number of cells released overtime was quantified using CyQUANT (DNA-based quantification) assay and by manual counting were shown in Figure 4b and Figure S7, respectively. As can be seen, it took about 2 h for the cells to sense and respond to the surface change and very few cells were actually released during this period. After 2 h, cells started to release from the surface at a relatively high rate, more than 60% of attached cells were released during the next 4 h. The cell release rate was slower after 6 h and the release rate was insignificant towards the end of the experiment (24 h). It should be noted that the viability of released cells was higher than 96% at all tested time points as quantified by Trypan blue assay (Figure 4c), indicating no significant cytotoxicity resulting from the change in Polymer **3** conformation. In addition, the metabolic activity of remaining attached cells was measured with Presto blue assays, which indirectly quantified the number of remaining attached cells on the coated surface. As shown in Figure 4d, the percentage of remaining attached cells decreased with time during the first 6 h at 37 °C, which was in an agreement with the increased number of cumulatively released cells during the same period. However, a small increase of the number of attached cells was observed after incubation at 37 °C for about 10 h. It is likely that the remaining attached cells started to proliferate during this time, which explains the slight increase in Presto blue measurements towards the 24 h time point.

## 3. Discussion

**Figure 5:**
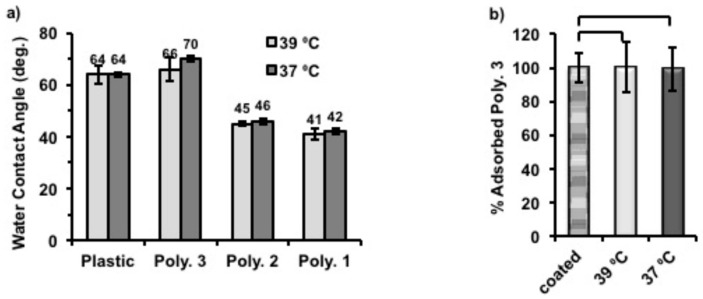
(**a**) Water contact angle measured on plastic coverslips with and without polymer coatings on top at 37 and 39 °C. Column graphic comparison of static water contact angles between differently coated samples measured with at least 4 repeats. (**b**) The percentage of Polymer **3** remained on the surface after culturing for 6 h at 37 and 39 °C compared to the amount of Polymer **3** right after coating at 39 °C overnight (name: coated) which was set to be 100% in this experiment (*n* = 3). Statistical significance was determined using one way ANOVA, α = 0.05. Error bars indicate standard deviation (SD).

In order to probe the surface hydrophobicity change after coating with polymers, plastic coverslips were again coated with Polymer **1**–**3** using the same protocol described above. Static water contact angles were then measured for each sample at 39 and 37 °C. (Figure 5a) The static water contact angles of coated substrates decreased, with the increase of hydrophilicity of coating polymers (Polymer **1** ˃ Polymer **2** ˃ Polymer **3**). Thus, the surfaces became more hydrophilic with Polymer **1** coatings than when coated with Polymer **3**, which is also in accord with their structural compositions. Particularly, the static water contact angle of Polymer **3**-coated surface showed the closest values (66° at 39 °C, and 70° at 37 °C) to uncoated plastic (around 64° at both temperatures) which were found to be within the range that gives maximum adhesion of fibroblasts [31,32], while those of Polymer **1** and Polymer **2** were between 40° and 50° at both temperatures. Also, we found that Polymer **3**-coated surfaces became more hydrophobic (4° higher) when the assay temperature was changed from 39 to 37 °C. Although the same response to temperature was also observed for Polymer **1** and **2** coated surfaces, the contact angle difference for them was only 1° between two temperatures. It was anticipated that the phase transition temperatures of Polymers **1** and **2** were lower or just in between the applied temperatures and the solubility of these polymers would not vary much due to such a small temperature change. However, the chains of Polymer **3** were expected to vary in conformation across the assay as the phase transition temperature of Polymer **3** was close 39 °C on cooling cycles. Therefore, we believe that this temperature related surface hydrophobicity change of Polymer **3** was the cause of cell release when altering the culture temperature. As expected, uncoated plastic coverslips used as control surfaces did not show temperature-dependent behavior.

To understand better the mechanism of temperature triggered cell release from Polymer **3** coated nTCP, the amount of Polymer **3** on each well after incubation at different temperatures was quantified by using a Pierce Quantitative Colorimetric Peptide Assay. In this experiment, the amount of Polymer **3** on each well immediately after coating was approximately 0.70 µg/cm^2^. Two coated plates were then incubated with 500 μL of deionised H_2_O in each well for 6 h at 37 °C and 39 °C, respectively. The remaining amount of Polymer **3** after this was quantified and compared to that of the initially coatings (0.70 µg/cm^2^ set to be 100%). As shown in Figure 5b, more than 99% of initially coated Polymer **3** remained on the surfaces after incubation at both temperatures, indicating that this polymer did not detach from the surface in aqueous media at both tested temperatures. The standard curve made to quantify the adsorbed polymers can be found in Figure S8.

To evaluate protein adsorption on polymer coated surfaces, nTCPs were used as the substrates for polymer coating and as a negative control. TCPs were used as positive control to compare the effect of polymer coating investigated. Polymer **4** was used as a non thermoresponsive control coating polymer. The protein resistance of Polymer **4** is from the low fouling non-ionic polyethylene glycol (PEG) at its surface. A relatively high concentration of (Monomeric red fluorescent protein1) mRFP1 solution (200 μg/mL) was used in this experiment in order to give readable fluorescent intensities of mRFP1 as its surface adsorption was low. Proteins were allowed to adsorb on surfaces over 24 h to ensure that the adsorption reached a maximum [33]. In order to evaluate the correlation between protein adsorption and its charge, two variant mRFP1s, with both positive (pI = 9.66) and negative (pI = 5.65) net overall charges in deionized H_2_O solution at pH 7, were incubated in coated wells at 37 °C for 24 h, and handling time thereafter was reduced as short as possible. The supernatants from each well were therefore removed after incubation. The plates were rinsed with copious amount of PBS twice and deionized water once to remove all the improperly adsorbed protein. The emission collection band width in our assay spanned the excitation wavelength, therefore the excitation wavelength was set up to be 620 nm, instead of 607 nm as reported elsewhere [34].

**Figure 6:**
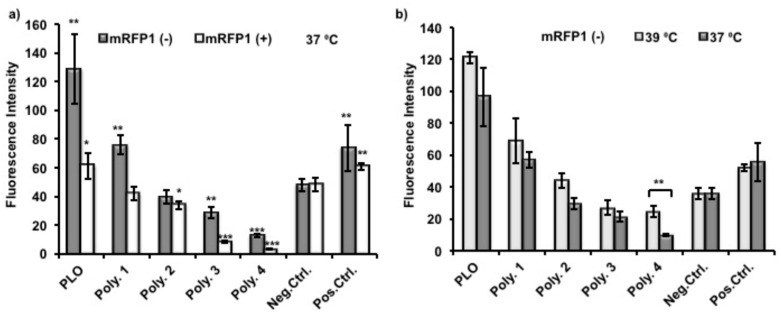
(**a**) Normalized fluorescence intensity of different polymer coated nTCP surfaces, uncoated nTCP (Neg. Ctrl.) and TCP (Pos. Ctrl.) surfaces adsorbed with mRFP1 (negative charge, pI = 5.65 and positive charge, pI = 9.66) after incubated at 37 °C for 24 h and (**b**) adsorbed with mRFP1 (negative charge, pI = 5.65) after incubation at 39 and 37 °C, respectively, for 24 h. A student t-test was used to compare the difference between the coatings and the uncoated nTCP (Neg. Ctrl.), or between the measurements at 39 and 37 °C (*n* = 3, * *p*  < 0.05, ** *p*  < 0.01, *** *p*  < 0.001).

As shown in Figure 6a, the fluorescence intensities from adsorbed protein were normalized and therefore giving the same readings of negative control between different experiments. Poly(l-ornithine) (PLO)-coated surfaces showed the highest fluorescence intensities from adsorbed mRFP1, and therefore indicated significant enhancement of protein adhesion compared to uncoated nTCP (negative control). The fluorescence intensities from adsorbed mRFP1 with either charge on Polymer **1** coated surfaces were higher than those with Polymer **2** coatings which were higher than those coated with Polymer **3**. These results suggest that the chemical modification, to some extent, conferred protein resistance to Polymers **1**–**3** compared to unmodified PLO. The more hydrophobic polymers with fewer primary amines demonstrated reduced protein attachment. The fluorescence intensities of Polymer **3** and **4** coated surfaces were significantly lower than those of uncoated nTCP (negative control) and TCP (positive control), indicating detectable protein resistance capability. However, this effect was less significant on negatively charged mRFP1. In all cases, lower fluorescence intensities were observed from adsorbed positively charged mRFP1 than that from negatively charged mRFP1. We expected that the amino residues in the polymers were partially protonated at this experimental condition (pH 7.4), and the charge-to-charge repellence between coating polymers and positively charged protein would enhance the overall effect of protein resistance. The temperature response experiment was performed by using negatively charged mRFP1 at the two practical temperatures. Although the fluorescence intensities were apparently lower, meaning less proteins were adsorbed, when the temperature was decreased from 39 to 37 °C, only Polymer **4** showed significant differences in fluorescence (Figure 6b).

**Figure 7:**
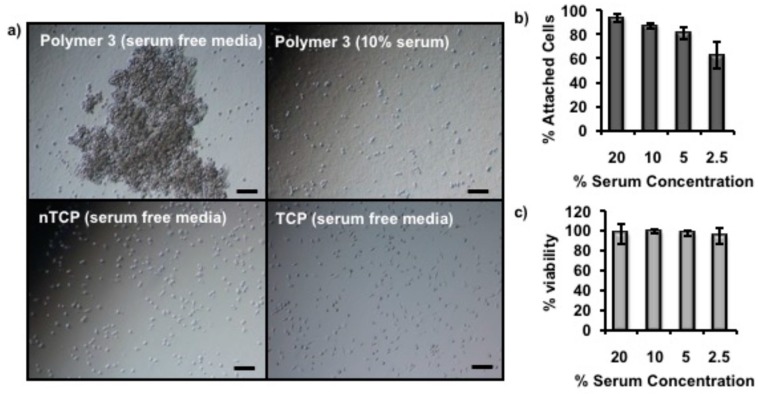
(**a**) Microscope images of 3T3 cells taken on Polymer **3** coated nTCP cultured at 39 °C for 5 h in the absence and presence of serum (10%) in the media, and on uncoated nTCP and TCP in the absence of serum in the media. Big cell aggregate was observed on Polymer **3**-coated nTCP without serum in the media, while cell attachments were recorded in all the other three circumstances (scale bar = 100 µm). (**b**) The column graph of the percentage of attached cell with different concentrations of serum in the media indicates serum concentration related cell attachment on Polymer **3** coated nTCP. (**c**) The viability of the cells through this experiment with tested serum concentrations was measured by counting the dead number of cells dyed with Trypan blue (*n* = 3).

Serum plays an important role in cell attachment as the adhesion proteins can be adsorbed onto appropriate plastic surfaces and facilitate attachment [35]. To further understand the mechanism of the cell attachment on Polymer **3** coated nTCP, a serum depletion experiment was carried out with 3T3 cells cultured at 39 °C for 5 h. The cell attaching time was shortened to be 5 h rather than 18 h in this experiment. This is because the cells may start to die in the absence of serum in the culture media for long incubation time. As shown in microscopy images in Figure 7a, a large cell aggregate was observed on Polymer **3** coated nTCP with no serum in the media, while individual cell attachments were observed with 10% serum in the media on the same surface during this incubation time. Interestingly, more regular cell attachment was shown on both uncoated nTCP and TCP even in the absence of serum in the media as shown in Figure 7a. Further experiments were performed with 3T3 cells in the same condition with different concentrations of serum in the media, the results of which are shown in Figure 7b. These data indicate that the percentage of attached cells was reduced with diluted serum concentration in the media. In all cases, more than 94% of the cells were viable during this experiment (Figure 7c).

We found overall that the cell attachment was serum concentration-dependent and considerable cell aggregation was observed in the absence of serum in the media. Therefore, we believe that serum adsorption to the polymer surfaces was important in the increase in 3T3 cell attachment. Although the results of protein adsorption experiments (Figure 6) showed that fewer proteins were adsorbed on the Polymer **3** coated nTCP compared to the uncoated nTCP, there were still quantifiable proteins adsorbed on the coated surface, especially the negatively charged proteins which established electrostatic interaction with positively charged coating Polymer **3**. A key example of such a protein is bovine serum albumin (BSA) which is a major component of fetal calf serum (FCS) and exhibits a net negative charge at physiological pH [36]. Additionally, different types of proteins respond to the surface hydrophobicity differently as reported previously [32], and thus we intend to test the adsorption behavior of more specific examples of proteins in future experiments. However, from the results presented so far, we believe that the rich variety of proteins in FCS is essential to successful cell attachment on the proposed coated surfaces.

## 4. Materials and Methods

### 4.1. Materials

Poly(l-ornithine) hydrobromide (*M*_w_: ˃ 100,000), Poly(l-lysine) hydrobromide (*M*_w_: 15,000–30,000), methoxypolyethylene glycol succinate *N*-hydroxysuccinimide (mPEG-*NHS*, *M*_w_: 5000, 90%), *N*-succinimidyl *N*-methylcarbamate (97.0%), used as a safe methyl isocyanate substitute, and potassium cyanate (96%), sodium tetraborate (99%), imidazole buffer (1 M, pH 9–10), trifluoroacetic acid (TFA, 99%), phosphate buffered saline tablets and deuterium oxide (D_2_O; 99.9 atom% D) were purchased from Sigma Aldrich (Dorset, UK) and used as received. Solvents were obtained from Fisher Scientific (Loughborough, UK) and used as received. Deionized water was obtained from an Elga Pure Nanopore 18.2 MΩ water purification system (High Wycombe, UK). Aqueous HCl and NaOH solutions were used to adjust the solution pH as desired. Dialysis membrane with 1000 MWCO and 6000–8000 MWCO were purchased from Spectrumlabs (Rancho Dominguez, CA, USA). 

Dulbecco’s modified Eagle media (DMEM), 10% fetal calf serum (FCS), 1% antibiotic/antimycotic solution and 1% l-glutamine (2 mM) were purchased from Sigma Aldrich (Dorset, UK). Presto blue cell viability reagent was obtained from Invitrogen (Hemel Hempstead, UK). Trypan blue solution (0.4%, prepared in 0.81% sodium chloride and 0.06% potassium phosphate, dibasic) was purchased from Sigma Aldrich (Dorset, UK). CyQUANT™ NF cell proliferation assay kit (Invitrogen) was purchased from Fisher Scientific (Loughborough, UK). Monomeric red fluorescent protein1 (mRFP1) with either negative (pI: 5.65, *M*_w_: 25.4 kDa) or positive (pI: 9.66, *M*_w_: 29.4 kDa) charges at physiological pH were kindly provided by Dr. James Dixon from the same group. Pierce quantitative colorimetric peptide assay kit was purchased from ThermoFisher Scientific (Hemel Hempstead, UK).

### 4.2. Synthesis of Poly(l-ornithine)-co-poly(l-citrulline)(POC)—Polymer **1**

Poly(l-ornithine) hydrobromide (*M*_w_: ˃ 100,000, 84.0 mg, 0.43 mmol of l-ornithine) and potassium cyanate (43.7 mg, 0.52 mmol) was weighed in a glass vial, and dissolved in imidazole buffer solution (1 M, pH 7.0, 4 mL). The reaction was started by placing the sealed glass vial an oil bath at 50 °C and then kept stirring for 24 h. The reaction was stopped by cooling down to room temperature. The crude product was purified by dialysis against deionized H_2_O (0.1% trifluoroacetic acid, TFA) with 1000 MWCO dialysis membrane for 2 days. Then, water was removed by freeze-drying. The product was stored at −80 °C [26].

### 4.3. Synthesis of Methyl Isocyanate Modified Poly(l-ornithine)-co-Poly(l-citrulline) (Methyl Isocyanate Modified POC)—Polymer **2** and **3**

Typically, poly(l-ornithine) hydrobromide (*M*_w_: ˃ 100,000, 84.0 mg, 0.43 mmol of l-ornithine) and *N*-succinimidyl *N*-methylcarbamate (7.6 mg, 0.043 mmol) was weighed in a glass vial, and dissolved in sodium tetraborate buffer solution (50 mM, pH 8.5, 4 mL) with continuous stirring at room temperature for 24 h. Potassium cyanate (43.7 mg, 0.52 mmol) was then added in the reaction solution. The sealed glass vial was placed in an oil bath at 50 °C and kept stirring for another 24 h. The reaction was stopped by cooling down to room temperature. The crude product was purified by dialysis against deionized H_2_O (0.1% trifluoroacetic acid, TFA) with 6000–8000 MWCO dialysis membrane for 2 days. Then, water was removed by freeze-drying. The product was stored at −80 °C.

### 4.4. Synthesis of PEG Grafted Poly(l-lysine) (PEG-g-PLL)—Polymer **4**

Poly(l-lysine) hydrobromide (*M*_w_: 20,000, 21.0 mg, 0.10 mmol of l-lysine) and methoxypolyethylene glycol succinate N-hydroxysuccinimide (mPEG-NHS, *M*_w_: 5000, 55.8 mg, 0.01 mmol) was weighed in a glass vial, and dissolved in sodium tetraborate buffer solution (50 mM, pH 8.5, 1 mL) with continuously stirring at room temperature for 6 h. The reaction was stopped by adding a large volume of deionized H_2_O, and purified by dialysis against deionized H_2_O with 6000–8000 MWCO dialysis membrane for 2 days. Then, water was removed by freeze-drying. The product was stored at −80 °C [27].

### 4.5. Nuclear Magnetic Resonance (NMR) Spectroscopy

^1^H-NMR and ^13^C-Heteronuclear Single Quantum Correlation (^13^C-HSQC) spectroscopies were recorded with an average of 64 scans per spectrum at 70 °C in D_2_O using a Bruker AV400 spectrometer (Coventry, UK) fitted with a 5 mm auto-tunable broad-band (BBFO) probe. Spectra were analyzed with MestReNova 6.2 software (Mestrelab, Santiago de Compostela, Spain). Chemical shifts were recorded in ppm (δ).

### 4.6. Cloud Point Measurements by Ultraviolet-Visible (UV-Vis) Spectroscopy

Polymers were dissolved in PBS (100 mM, pH 7.4) to give a concentration of either 1 mg/mL or 0.2 mg/mL. For the UCST type thermal responsive polymers, heating was applied to dissolve the polymers. The solution was transferred into the cells immediately. Absorbance at 500 nm of the polymer solutions in a 10 mm quartz cell were recorded on Shimadzu UV-1650PC UV-visible spectrophotometer (Milton Keynes, UK) equipped with a Peltier temperature controller (Southampton, UK) at scanning rate of 1 °C/min either from 20 °C to 80 °C or reversely from 80 °C to 20 °C.

### 4.7. Preparation of Thermal Responsive Polypeptides Coated Non-Tissue Culture Plates

Polymers (1 mg/mL) in deionized H_2_O were ultra-sonicated at 70 °C for 15 min to give transparent solutions. Warm polymer solutions were sterilized by filtering through 0.22 µm membranes, and then transferred into 24-well non-tissue culture plate (300 µL for each well) immediately. The plate was then incubated at 39 °C overnight. The solutions were then removed. The coated wells were rinsed with PBS buffer once, and dried in the fume hood before use.

### 4.8. Time of Flight Secondary Ion Mass Spectrometry (ToF-SIMS)

ToF-SIMS data were collected using a ToF-SIMS IV instrument (ION-TOF GmbH., Münster, Germany) equipped with a bismuth liquid metal ion gun and a single-stage reflectron analyzer. Bi_3_^+^ primary ion energy of 25 kV and a pulsed target current of approximately 1.3 pA were used in this measurement. Low-energy electrons (20 eV) were used to compensate for surface charging due to the positively charged primary ion beam on the insulating surfaces. Rastered areas of 3 × 3 mm^2^ were analyzed at a resolution of 100 pixels per mm and 15 frames per patch. The total primary ion beam dose for each analyzed area was kept below 1 × 10^12^ ions per cm^2^, ensuring static conditions. Data acquisition and analysis were performed using IONTOF SurfaceLab6 software (IONTOF, Munster, Germany).

### 4.9. Cell Adhesion for Screening Coating Polymers

NIH-3T3 fibroblast cells were grown in DMEM with 10% FCS, 1% antibiotic/antimycotic solution and 1% l-glutamine (2 mM). Tissue culture-treated plates, non-tissue culture-treated plates, and either poly(l-lysine) or poly(l-ornithine) coated wells were used as controls. 3T3 cells were added (1 × 10^5^ cells per well), and incubated at 37 °C overnight to allow cells to attach. Microscope images were taken after incubation for 24 h. Alexa Fluor 488 Phalloidin (ThermoFisher Scientific, UK) that binds intracellular F-actin, was used to visualize the attached cells, and the microscope images were recorded again. After cell attachment in the polymer coated plates, Live/Dead^®^ Viability/Cytotoxity kit (Invitrogen) was used to differentiate live cells which take up the green fluorescent Calcein AM and dead cells which take up Ethidium homodimer-1. The cells were treated with the stain at 39 °C for 15 min and imaged using fluorescence microscope. The culture temperature was changed to 37 °C and the Live/Dead stained cells were imaged after 2 h.

### 4.10. In Vitro Cell Release Studies

3T3 cells were seeded (1 × 10^5^ cells per well) into Polymer 3 coated non-tissue culture treated 24-well plates with 500 µL of cell culture media in each well, and incubated at 39 °C overnight (16 h) to allow cells to attach. The unattached cells along with the cell culture media were removed from the wells. The wells were then refilled with the same volume (500 µL) of fresh cell culture media and incubated at 37 °C (cell release triggered). The metabolic activity of attached cells was measured by using Presto blue assay at every time point. The cell numbers were inferred from the standard curve experiment with cell numbers and fluorescence readings. The released cells in the supernatant was quantified by using CyQUANT™ (ThermoFisher Scientific, UK) NF cell proliferation assay kit Invitrogen, and the viability was tested by using Trypan blue assay. The cells that were released in to the supernatant were centrifuged, trypsinized and counted using Trypan blue in one set of experiments. The cells were treated with CyQUANT as described in the manufacturer’s protocol and the fluorescence intensity was measured in a microplate reader with excitation 485 nm and emission detection at 530 nm. The released cell numbers were determined using a standard curve by plotting the fluorescence unit against different cell numbers. The experimental design of the release study is given as a schematic in Figure S9. These steps were repeated at determined time points (e.g., 2 h, 4 h, 6 h, 12 h and 24 h) until no more cells were released into the supernatant.

### 4.11. Contact Angle (CA) Measurement

Static water contact angle measurements were made on each polymer coated surface using a CAM 200 Optical Contact Angle Meter (KSV Instruments Ltd., Helsinki, Finland). This instrument was fitted with a thermostated mental cell, connected to a refrigerated/heated bath circulator (Fisherbrand, UK) to maintain the temperature of the sample.

### 4.12. Quantification of Polymers Adsorbed on nTCP

Polymer **3** was coated on three non-tissue culture treated 24-well plates as described above. In one plate, the amount of coated Polymer **c** on each well was quantified by using a Pierce quantitative colorimetric peptide assay kit. The other two plates were then incubated with freshly added deionized H_2_O (500 µL per well) for 6 h at 37 °C and 39 °C, respectively. The remaining amount of Polymer **c** on each well in these two plates was then quantified.

### 4.13. Adsorption of Proteins to Polymer Coated Surfaces

The method to prepare red fluorescent mRFP1 protein was adapted from Campbell et al. [34]. To determine the protein adsorption to surfaces, mRFP1 was diluted to 200 μg/mL with PBS buffer (100 nM, pH 7.4). This protein solution was added to the polymer-coated 24-well plates (300 μL per well). The plates were then incubated at 39 °C, 37 °C or room temperature for 24 h. The wells were rinsed with PBS buffer (300 μL) twice and then deionized water (300 μL) once. The adsorption of mRFP1 on surfaces was quantified by the reading of fluorescent intensity (excitation wavelength 584 nm, emission wavelength 620 nm) on a Tecan (Theale, UK) plate reader (infinite M200).

### 4.14. Serum Depletion Experiment

3T3 cells were seeded (1 × 10^5^ cells per well) into Polymer **3**-coated non-tissue culture treated 24-well plates with 500 µL of cell culture media (containing 20%, 10%, 5%, 2.5% and 0%, *v*/*v* FCS, respectively) in each well, and incubated at 39 °C for 5 h. Microscope images were then taken. The number and viability of the cells in the supernatant was then counted using a Haemocytometer combined with Trypan blue assays.

### 4.15. Statistical Analysis

Statistical comparisons were carried out using GraphPad Prism (6.01, GraphPad Software, Inc. San Diego, CA, USA). The statistical significance was determined using a one-way analysis of variance (ANOVA) followed by Dunnet’s test for viability and cell release experiments; Student’s t test was carried out for protein adsorption experiments. Results were considered significant at *p* < 0.05.

## 5. Conclusions

UCST-type thermal responsive polypeptides (TRPs) with phase transition temperatures around 37 °C in PBS buffer (pH 7.4, 100 mM), were prepared from commercial available poly(l-ornithine) hydrobromide. These positively charged materials were readily coated on non-tissue culture treated plates (nTCP). Initial screening experiments performed with 3T3 cells demonstrated that only Polymer **3** coated surface resisted cells from adhesion at 37 °C, but allowed them to attach at close to body temperature (39 °C) during 24 h incubation. Polymer **3**, showing a phase transition around 39 °C (1 mg/mL) in PBS buffer, was selected for further studies. The cell release from Polymer **3** coated surface was triggered when switching the culture temperature to 37 °C. Approximate 65% of the attached cells were released from the surface within 6 h, and more than 96% of the released cells retained their viability. The water contact angle measurements performed at 39 and 37 °C demonstrated the surface hydrophobicity change of Polymer **3** in response to the applied temperatures, which we believe is the reason for the cell release. Also, the amount of polymers on the surfaces after culturing at different temperatures was quantified to be the same as that measured immediately after the coating process, which means that the cell release was not due to the loss of coating polymers. In addition, serum depletion experiments demonstrated that cell adhesion on TRPs coated nTCP was strongly related to the concentration of serum, which improved the cell adhesion on the coated surfaces. The new TRP coatings therefore were likely to have acted via a ‘hybrid’, protein to surface interaction, which mediated cell attachment and release, as the underlying polymer surface changed conformation and accordingly displayed any surface-adsorbed proteins differently. The 3T3 cells were released with high viability by the temperature changes used (around body temperature), and therefore we suggest these surfaces should have potential applications in cell-based therapies where ‘switching’ of cell attachment is required.

## Figures and Tables

**Table 1:** Summary of the structural composition of polymers synthesized and their phase transition temperatures in phosphate-buffered saline (PBS) buffer (100 mM, pH 7.4).

Product Code	Structural Composition (mol.%)				Phase Transition Temperature (*T*_p_, °C)			
	–NH_2_	–NHCONH_2_	–NHCONHCH_3_	PEG	1 mg/mL		0.2 mg/mL	
					Cooling	Heating	Cooling	Heating
Polymer **1**	10	90	0	-	32	37	30	35
Polymer **2**	<10	>80	10	-	38	42	35	39
Polymer **3**	<<10	<90	10	-	39	N/A	36	N/A
Polymer **4**	90	-	-	10	-	-	-	-

## Supplementary Materials

The following are available online at www.mdpi.com/1996-1944/11/1/95/s1. Figure S1: ^1^H-NMR(400 Hz, D_2_O) spectrum for Polymer **1** at 70 °C, Figure S2: ^1^H-NMR(400 Hz, D_2_O) spectrum for Polymer **2** at 70 °C, Figure S3: ^13^C-HSQC (400 Hz, D_2_O) spectrum for Polymer **3** at 70 °C, Figure S4: ^1^H-NMR(400 Hz, D_2_O) spectrum for Polymer **4** at room temperature, Figure S5: ToF SIMS spectra and image of signals from specific chemical structures for Polymer **1**, **2**, **3**, **4**, PLO, PLL coated plastic surface and Blank control, Figure S6: Live/Dead staining of cells on Polymer 3. Figure S7: Percentage of cells, in relation to the total number of cells seeded, which were released over differential times (2, 4, 6, 12, 24 h) from the polymer-coated surface at the polymer phase-transition temperature of 37 °C, determined using manual counting with Trypan blue, Figure S8: Standard curve for Pierce Quantitative Colorimetric Peptide Assay, Figure S9: Schematics of cell release study experimental design.
