# Supplementary material for: Post-Modified Polypeptides with UCST-Type Behavior for Control of Cell Attachment in Physiological Conditions
Source: Materials (Basel). 2018 Jan 9;11(1):95. doi: 10.3390/ma11010095 (PMC5793593; doi:10.3390/ma11010095)
Supplement: Supplementary file 1 [file materials-11-00095-s001.pdf]

Supplementary Materials: Post-Modified Polypeptides with UCST-Type Behavior for Control of Cell Attachment in Physiological Conditions

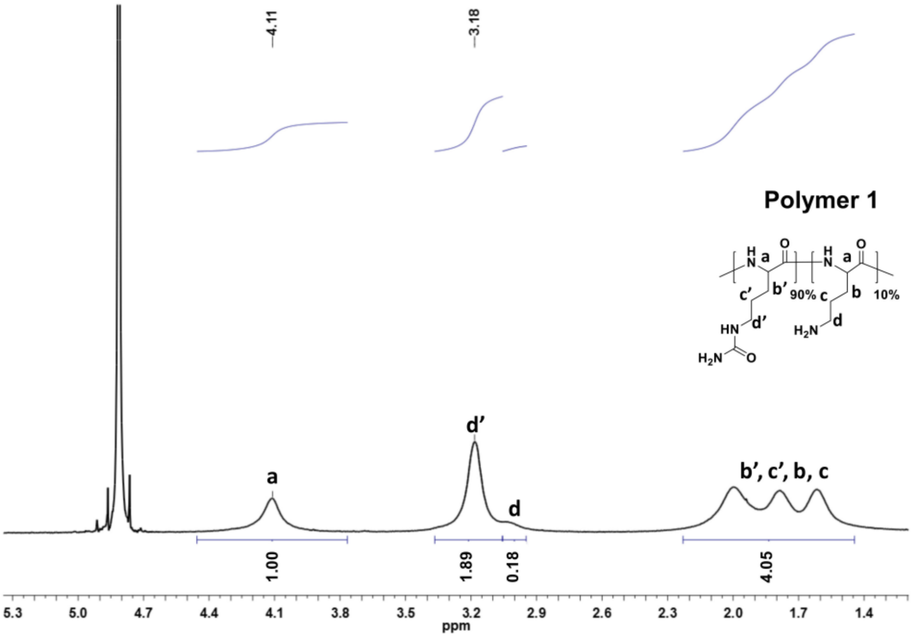

Figure S1. ¹H-NMR (400 Hz, D_2O) spectrum for Polymer **1** at 70 °C.

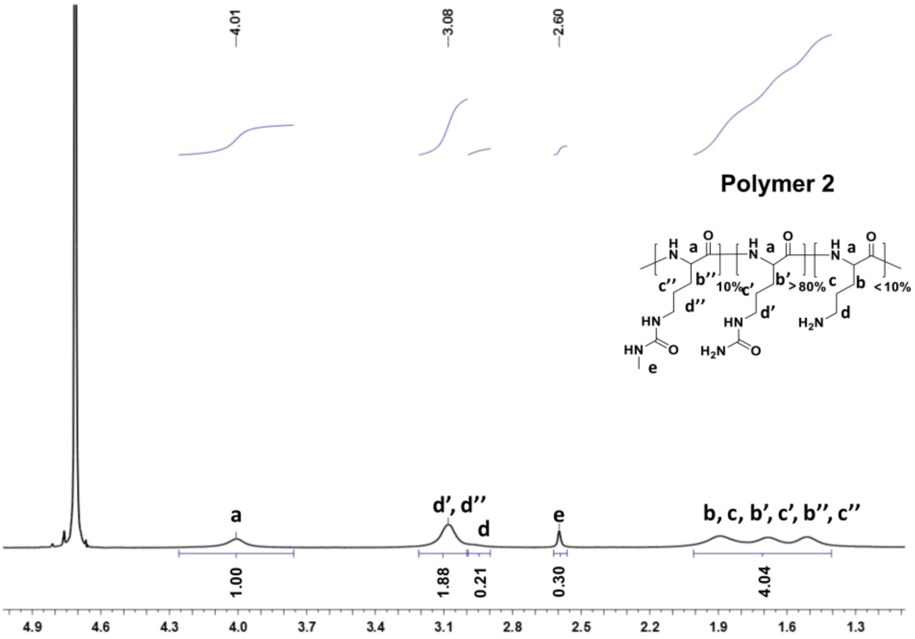

Figure S2. ¹H-NMR (400 Hz, D_2O) spectrum for Polymer **2** at 70 °C.

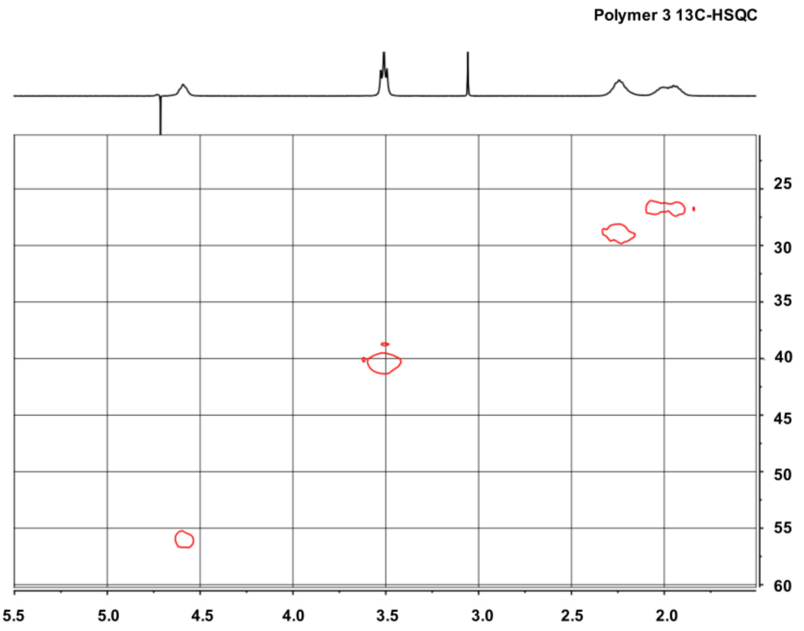

Figure S3. ^{13}C-HSQC (400 Hz, D_2O) spectrum for Polymer **3** at 70 °C.

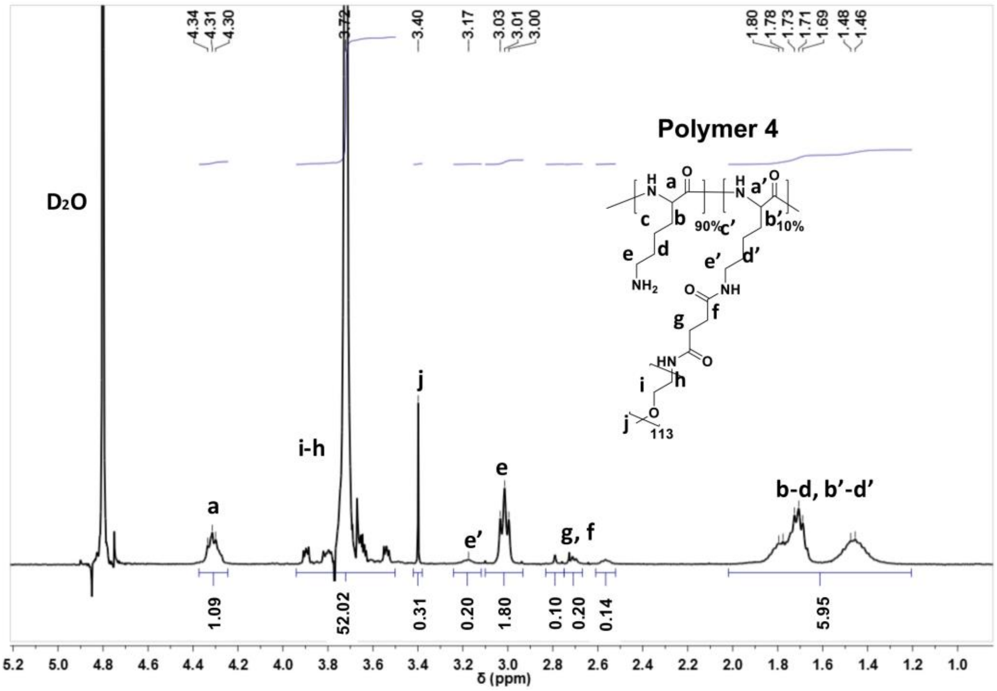

Figure S4. ^{1}H-NMR (400 Hz, D_2O) spectrum for Polymer **4** at room temperature.

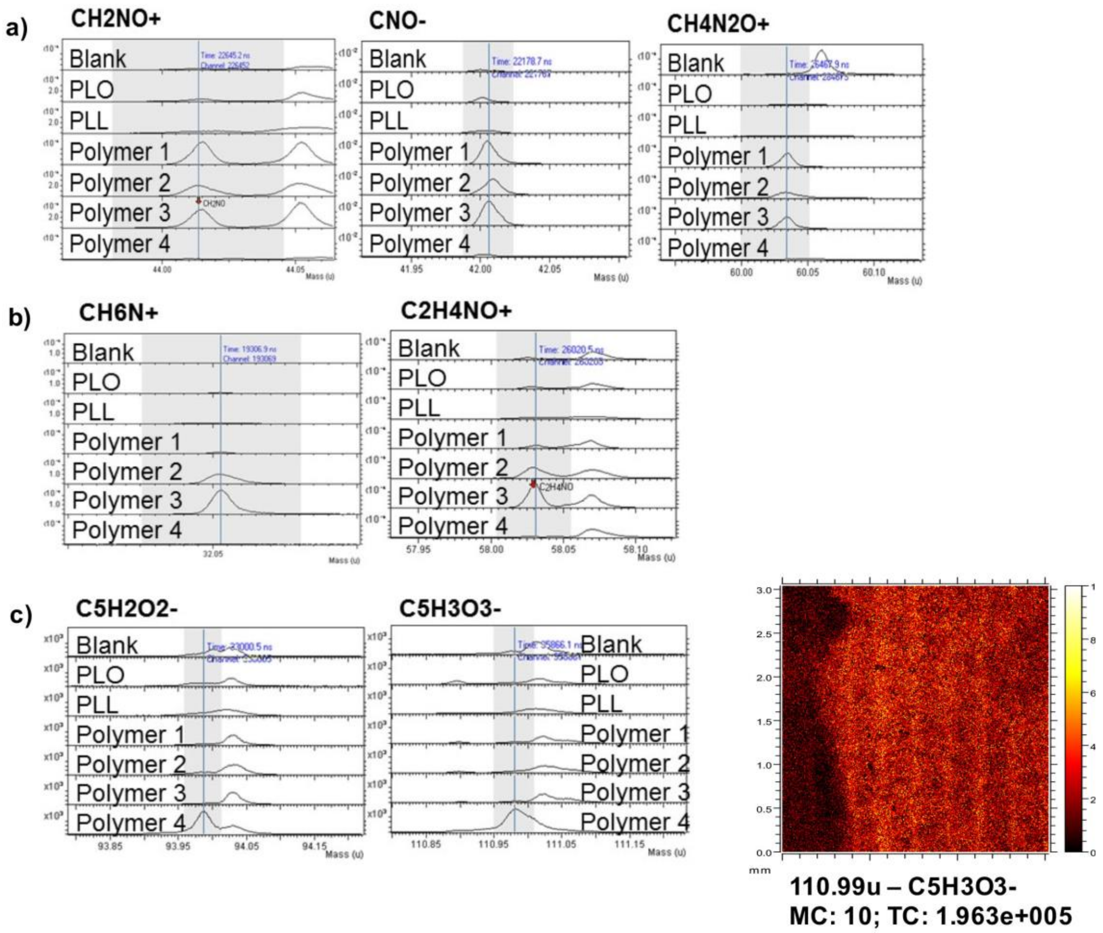

Figure S5. ToF SIMS spectra and image of signals from specific chemical structures for Polymer **1**, **2**, **3**, **4**, PLO, PLL-coated plastic surfaces, and Blank control. (**a**) Signals from ureido modification; (b) Signals from MIT modification; (c) Signals from PEG modification.

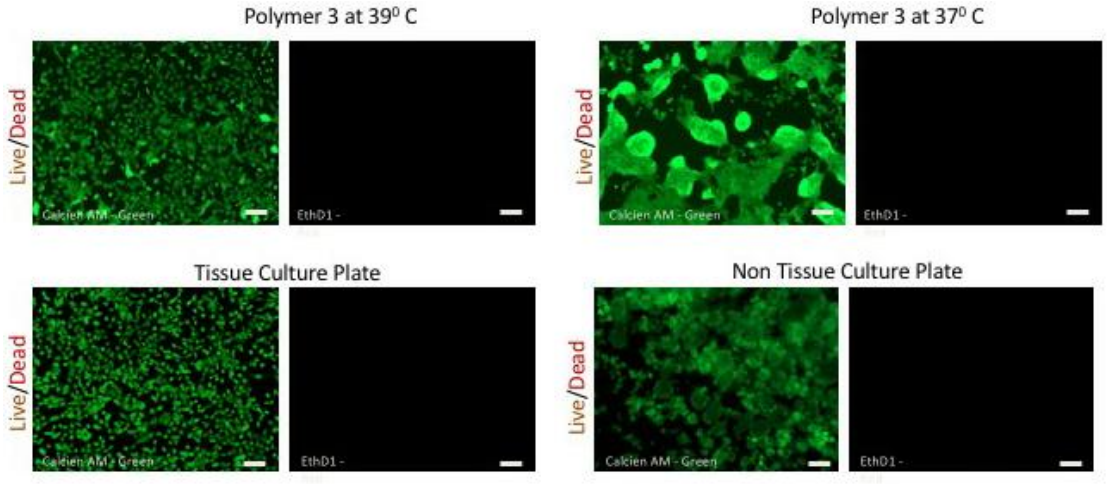

Figure S6. Live/Dead assays of cells attached on Polymer 3 coated surface, Tissue culture and non-Tissue culture plates at 39 °C after overnight incubation. The released cells from the Polymer 3 were imaged after 2 h incubation at 37 °C.

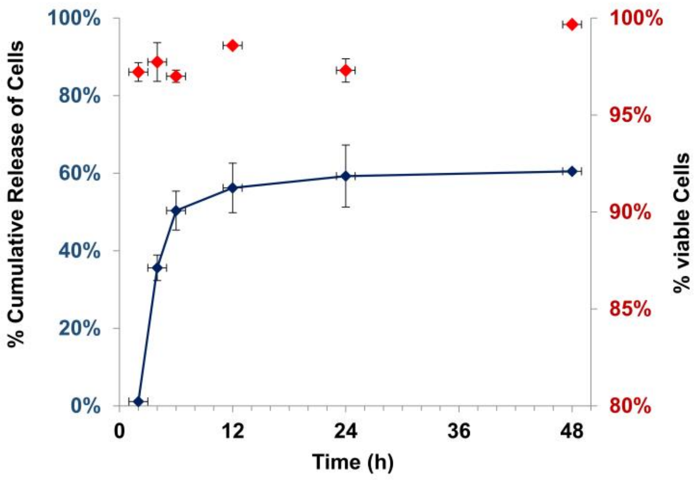

Figure S7. Percentage of cells, in relation to the total number of cells seeded, which were released over differential times (2, 4, 6, 12, 24 h) from the polymer-coated surface at the polymer phase-transition temperature of 37 °C, determined using manual counting with Trypan blue.

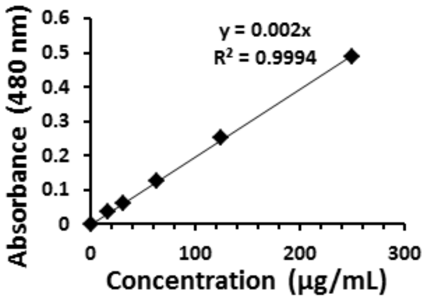

Figure S8. Standard curve for Pierce Quantitative Colorimetric Peptide Assay.

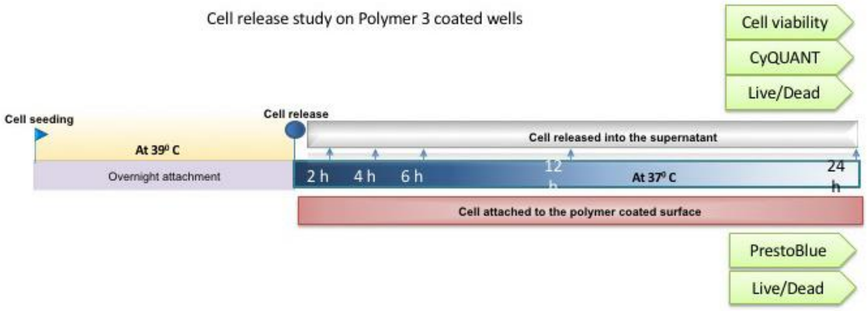

Figure S9. Schematic of the cell release study. After culturing the cells at 39 °C overnight on Polymer **3**-coated surfaces, the cells were transferred to solutions at 37 °C. The released cells were analyzed using either Trypan blue + counting or with CyQUANT at each of the time points. The attached cells were analyzed using Presto blue at the respective time points. A Live/dead assay was performed to visualize cell release.
